# Is Anti-NXP2 Autoantibody a Risk Factor for Calcinosis and Poor Outcome in Juvenile Dermatomyositis Patients? Case Series

**DOI:** 10.3389/fped.2021.810785

**Published:** 2022-02-23

**Authors:** Natasa Toplak, Pallavi Pimpale Chavan, Silvia Rosina, Tomas Dallos, Oz Rotem Semo, Cassyanne L. Aguiar, Raju Khubchandani, Angelo Ravelli, Anjali Patwardhan

**Affiliations:** ^1^Department of Allergology, Rheumatology and Clinical Immunology, Faculty of Medicine, University Children's Hospital, University Medical Centre, Ljubljana, Slovenia; ^2^Pediatric Rheumatology, NH SRCC Children's Hospital, Mumbai, India; ^3^Pediatric Rheumatology, IRCCS Istituto Giannina Gaslini, Genoa, Italy; ^4^Department of Paediatrics, Comenius University Medical School, National Institute of Children's Diseases, Bratislava, Slovakia; ^5^Section of Pediatric Rheumatology, University of Chicago Medical Center, Chicago, IL, United States; ^6^Pediatric Rheumatology, Eastern Virginia Medical School (EVMS), Children's Hospital of The King's Daughters, Norfolk, VA, United States; ^7^Direzione Scientifica, IRCCS Istituto Giannina Gaslini, Genoa, Italy; ^8^Dipartimento di Neuroscienze, Riabilitazione, Oftalmologia, Genetica e Scienze Materno-Infantili (DiNOGMI), Universita Degli Studi di Genova, Genoa, Italy; ^9^Pediatric Rheumatology, University of Missouri, School of Medicine, Columbia, MO, United States

**Keywords:** juvenile dermatomyositis, anti-NXP2 autoantibodies, disease outcome, treatment, risk factors

## Abstract

Juvenile dermatomyositis (JDM) has a wide spectrum of clinical presentations. In the last decade, several myositis-specific antibodies have been identified in patients with JDM and connected with specific organ involvement or specific clinical picture. It has been published that the presence of anti-NXP2 autoantibodies presents a risk for calcinosis in patients with JDM. We aimed to investigate the prevalence of calcinosis and response to the treatment in JDM patients with anti-NXP2. In a retrospective, multinational, multicenter study, data on 26 JDM (19 F, 7 M) patients with positive anti-NXP2 were collected. The mean age at disease presentation was 6.5 years (SD 3.7), the median diagnosis delay was 4 months (range 0.5–27 months). Patients were divided into two groups (A and B) based on the presence of calcinosis, which occurred in 42% of anti-NXP2 positive JDM patients (group A). Four patients already had calcinosis at presentation, one developed calcinosis after 4 months, and 6 developed calcinosis later in the disease course (median 2 years, range 0.8–7.8). The differences in laboratory results were not statistically significant between the groups. The mean age at disease presentation (5.2/7.5 years) trended toward being younger in group A. Children with calcinosis were treated with several combinations of drugs. In four cases, rituximab and, in one case, anti-TNF alpha agents were used successfully. Disease outcome (by evaluation of the treating physician) was excellent in four, good in two, stable in two, and poor in three patients. None of the patients from group B had a poor disease outcome. In conclusion, JDM patients with anti-NXP2 are prone to develop calcinosis, especially if they present with the disease early, before 5 years of age. The development of calcinosis is associated with worse disease outcomes. The combination of several immunomodulatory drugs and biologic drugs can stop calcinosis progression; however, there are no evidence-based therapies for treating calcinosis in JDM patients.

## Introduction

Juvenile dermatomyositis (JDM) has a spectrum of clinical presentations, from mild forms of myositis and skin changes with a monocyclic course to very severe forms with vital organ involvement and late complications, including calcinosis and lipodystrophy (1). In the last decade, there has been a development in the determination of myositis specific autoantibodies (MSA) connected with distinct clinical phenotypes of JDM (2). Among different MSA, anti-NXP2 and anti-PM/Scl are most commonly associated with calcinosis in JDM and the adult form of dermatomyositis. In recently published studies, TIF1γ and MDA5 MSA were also risk factors for calcinosis (3, 4). Calcinosis is one of the major complications of JDM and is found in 20–40% of patients (5, 6). It has been published that young age at disease presentation, regardless of MSA, presents a risk for calcinosis in patients with JDM (7). On the contrary, study in 78 patients showed that younger patients had fewer typical findings at diagnosis and a milder disease course with shorter use of corticosteroids and immunosuppression. MSA were not analyzed in this study (8). However, the presence of anti-NXP2 increases the risk of calcinosis across all ages (7). MSA are also associated with disease severity, worse functional status, and persistent disease activity (9).

The study aimed to collect data on JDM patients with positive anti-NXP2 and analyze the risk for calcinosis, response to treatment, and outcome in the group of patients with calcinosis.

## Methods

### Study Design and Study Population

The study design was a retrospective multinational, multicenter study on patients with JDM with positive anti-NXP2. The main objective was to study if anti-NXP2 is connected with calcinosis in patients with JDM and the most effective treatment approach in JDM patients with calcinosis.

### Data Collection

We contacted pediatric rheumatology centers through international online pediatric rheumatology forums and personal contacts. Nine centers responded (4 from Europe, 4 from USA and 1 from India). Seven centers participated in a study (3 from Europe, 3 from USA and 1 from India). They included all their patients with andi-NXP2.

Patients' data were collected in a shared excel table. Data on gender, race, age at disease onset and age at disease diagnosis, age at inclusion to the study, clinical picture (skin changes, myositis, arthritis, pulmonary and gastrointestinal involvement), muscle function tests, laboratory results, data on treatment and outcome of disease were collected. Laboratory investigations were performed in each participating centre.

### Statistical Analysis

The statistical analysis was performed with IBM SPSS (software version 22), using Student's two-tailed *t*-test (equal variances assumed; Levene's test for equality of variances) for continuous variables (independent samples *t*-test for comparison of two groups and paired-samples *t*-test for comparison of variables before and after treatment) and Fischer's exact test for categorical variables. Statistical significance was set at *p* < 0.05.

Parents of included patients signed informed consent and/or Institutional Review Board (IRB) approval was obtained where needed.

## Results

The patient's characteristics are presented in Table 1.

**Table 1:** Clinical and laboratory characteristics of 26 JDM patients with anti-NXP2 autoantibodies: *p* values comparing Group A and B were non-significant for all variables.

	**All**	**Group A**	**Group B**
Number of patients	26 (19F/7M)	11 (10F/1M)	15 (9F/6M)
Ethnicity	14c, 8as, 3aa, 1h	5c, 4as, 2aa	9c, 4as, 1aa, 1h
Mean age at disease presentation (y)	6.5 (SD 3.7)	5.2 (SD 2.9)	7.5 (SD 3.8)
Age at study inclusion (y)	11.1 (SD 4.6)	11.5 (SD 4.2)	10.8 (SD 4.8)
Median diagnosis delay (mo)	4 (range 0.5–27)	4 (range 0.5–24)	3.6 (range 0.5–27)
Myositis (%)	25 (96)	11 (100)	14 (93)
Gottron's papule (%)	25 (96)	11 (100)	14 (93)
Typical rash (%)	25 (96)	11 (100)	14 (93)
Lipodystrophy (%)	5 (19)	4 (36)	1 (6)
Skin ulcerations (%)	4 (15)	3 (27)	1 (6)
Polyarthritis (%)	3 (11)	2 (18)	1 (6)
Gut involvement (%)	3 (11)	2 (18)	1 (6)
Lung involvement (%)	3 (11)	1 (9)	2 (13)
ESR (mean, mm)	16.9 (SD 12.1)	13.8 (SD 8.9)	19.3 (SD 13.8)
Platelet count (mean,10^9^/L)	281 (SD 91.7)	306.5 (SD 85.8)	258.4 (SD 94.7)
CK (mean, U/L)	1,700.3 (SD 2,508)	1,548.6 (SD 2,397.7)	1,811.6 (SD 2,664.9)
AST (mean, U/L)	107.2 (SD 99.4)	111.4 (SD 111.3)	103.8 (SD 93.1)
ALT (mean, U/L)	59.4 (SD 65.2)	72.2 (SD 87.5)	50.8 (SD 46.6)
LDH (mean, U/L)	909.7 (SD 863.2)	1,048.3 (SD 1,037.3)	808.0 (SD 732.1)
IgG (mean, g/L)	10.8 (SD 3.9)	11.8 (SD 3.7)	9.9 (SD 4.1)
ANA (number of positive patients)	21 (80%)	9 (81%)	12 (80%)
MDA-5	1	1	0
PM-SCL100	2	1	1
Mi-2beta	1	0	1
SRP	1	0	1

*Group A—patients with calcinosis, Group B—patients without calcinosis*.

*F, female; M, male; y, year; mo, month; c, Caucasian; as, Asian; aa, African-American; h, Hispanic*.

We collected 26 patients (19 F, 7 M) with JDM and positive anti-NXP2. Fourteen patients were caucasion, eight Asian, three African-American and one Hispanic ethnicity. The mean age at disease presentation was 6.5 years (SD 3.7), the median diagnosis delay was 4 months (range 0.5–27 months). Patients were divided into two groups (A and B) based on the presence of calcinosis. In the disease course, 11 patients (42%), 10 female and 1 male, developed calcinosis (group A). Four patients already had calcinosis at presentation, one developed calcinosis after 4 months, and six developed calcinosis later in the disease course (median 2 years, range 0.8–7.8). Four patients developed lipodystrophy in group A (1 in group B). In group A, three patients developed skin ulcerations (1 in group B), two patients had polyarthritis (1 in group B), two patients had gut involvement (1 in group B), and one patient had lung involvement (2 in group B).

The mean age at disease presentation (5.2/7.5 years) and mean CK level (1,548.6/1,811.6 U/L) trended toward lower levels in group A. The platelet count (306.5/258.4 10^9^/L), and mean values of AST (111.4/103.8 U/L), ALT (72.2/50.8 U/L), LDH (1,048.3/808 IU/L), and IgG (11.8/9.9 g/L) trended toward higher levels in in group A. However, the differences were not statistically significant. ANA antibodies were positive in 9/11 in group A and 12/15 in group B. Beside anti-NXP2 other MSA were also found. One patient in group A was positive for anti-MDA5, another patient in group A and in one patient in group B were positive for anti- PM/Scl-100 and one patient in group B was positive for anti-Mi-2beta and anti- SRP. The data on muscle strength measurement (MMT/CMAS) were available only in a few patients.

Treatments used for patients with calcinosis included methotrexate and glucocorticosteroids (GCS) (all patients), hydroxychloroquine (9), IVIG (7), cyclosporine (4), bisphosphonate (4), MMF (5), rituximab (4), cyclophosphamide (1), abatacept (1) and TNFα blocker (1).

Disease outcome (by evaluation of the treating physician) was excellent in 4, good in 2, stable in 2, and poor in 3 patients. None of the patients from group B had a poor disease outcome. Patients with excellent disease outcomes from group A were treated with GCS and methotrexate (4), hydroxychloroquine (3), IVIG (1), and cyclosporine (1). Out of two patients who had good outcomes, one was treated additionally with MMF, bisphosphonates, and rituximab, and the second was treated with cyclophosphamide and rituximab. One patient with calcinosis at the presentation (age 4 years) was also treated with anti-TNFα therapy, which stopped progression and partially dissolved the calcinosis. After 2 years of anti-TNFα therapy, the calcinosis started to progress, and the treatment was changed to MMF and rituximab, which stopped the progression of calcinosis.

Muscle enzymes and other laboratory parameters were significantly improved in all patients following treatment at the end of observation period (mean observation time 4.2 years) in a whole group of patients. There were no significant differences between the patients with or without calcinosis (Table 2). The data for comparison were available for majority of patients (AST 22/26, ALT 21/26, LDH 20/26, CK 22/26). Results of muscle-functional testing were available for only a few patients, so a statistical analysis was not possible.

**Table 2:** Laboratory results for AST, ALT, LDH and CK before and after treatment in 26 JDM patients with anti-NXP2 autoantibodies.

	**Before treatment (mean)**	After treatment^*^(mean)	***p***
AST (mean, U/L)	112.2	33.9	0.003
ALT (mean, U/L)	64.1	29.3	0.045
LDH (mean U/L)	901.7	314.9	0.011
CK (mean (U/L)	1,845.3	194.6	0.006

* *At data collection; mean observation time before, at diagnosis, and after treatment is 4.2 years*.

*Normal values: AST <37, ALT <35, LDH <245, CK <185 U/L*.

The clinical characteristics, treatment and outcome of patients with calcinosis are presented in Table 3.

**Table 3:** Clinical characteristics of calcinosis, treatment and outcome of patients with calcinosis.

	Age^*^/Sex	**Time of onset/Description of calcinosis**	**Therapy**	Outcome^**^
Patient 1	4/F	At disease presentation/2 solitary small nodules- left cubital fossa with lipodystrophy and one in calf muscles, relapses and progression of small nodules at 6 and 9 years after disease onset-arms, neck, legs; severe calcinosis on right side of the neck with lipodystrophy	Pulse MP, Mtx, CsA, HCQ, MMF, pamidronate, IVIG, anti-TNF alpha, rituximab	Stable
Patient 2	8/F	At disease presentation/solitary nodule on thigh, later progressed into diffuse “calcinosis universalis”—multitude of tiny nodules in the subcutis of upper and lower extremities, genital area, elbow extensor surfaces, 1 at the volar surface of the right forearm with exulceration	Pulse MP, Mtx, HCQ, MMF, pamidronate, IVIG, rituximab	Good
Patient 3	2/F	After 4 years and 10 months of disease duration/very discrete, only on tip of 5th finger of the right hand	Pulse MP, Mtx	Excellent
Patient 4	10/F	After 1 year disease duration/dorsum of right thigh, left distal arm (near cubital fossa)	Pulse MP, Mtx, HCQ, IVIG	Excellent
Patient 5	2.4/F	After 10 months of disease duration/ureteral wall (bilaterally, with ureteral stenosis) and left elbow	Pulse MP, Mtx, CsA, HCQ, IVIG	Good
Patient 6	8.7/F	After 2 years of disease duration/lateral part of left thigh and right gluteal region	Pulse MP, Mtx, CsA, CYC, HCQ, rituximab	Excellent
Patient 7	3.7/M	After 7 years and 10 months of disease duration/small nodules on knuckles	Pulse MP, Mtx, HCQ	Excellent
Patient 8	4.2/F	after 2 years and 2 months of disease duration/right gluteal region, left side neck, left scapular region and left elbow	Oral GCS, Mtx, HCQ, MMF, pamidronate	Stable
Patient 9	3.2/F	After 4 months of disease duration/vulvar region, right eyelid, left popliteal region along the tendons, right sacral region, left Achiles tendon, b/l gluteal region, left scapula, superficial plaque like/nodular—along thigh and shin (b/l), left lateral malleoli	Oral GSC, Mtx, CsA, MMF, pamidronate, IVIG	Poor
		No new lesions when on IVIG and Pamidronate infusions, new lesions on stopping IVIG and Pamidronate infusions		
Patient 10	2/F	At disease presentation/upper and lower legs bilaterally, buttocks and upper arms	Pulse MP, Mtx, HCQ, IVIG	Poor
Patient 11	9.5/F	At disease presentation/first as nodules, later became extensive sheets with oozing skin breaches; arms, legs, trunk	Pulse MP, Mtx, HCQ, MMF, IVIG, rituximab, orencia	Poor

*M, male; F, female; MP, methylprednisolone; GCS, glucocorticosteroids; mtx, methotrexate; CsA, cyclosporine; HCQ, hydroxychloroquine; MMF, mofetil mycophenolate; IVIG, intravenous immunoglobulins; CYC, cyclophoyphamide*.

* *Age at disease onset*,

** *Evaluation by the treating rheumatologist, Kendall and CMA available only in few patients*.

## Discussion

In this study we collected data about the subgroup of JDM patients with anti-NXP2 autoantibodies. To the best of our knowledge, we present the largest group of JDM patients with anti-NXP2. In the studies published so far, these patients present about 16–20% of all patients with JDM and develop calcinosis in about 40–50% (3, 4). In the published literature, the risk of calcinosis is estimated to be about 25–30% in JDM patients (10, 11). In our study, 42% of JDM patients with anti-NXP2 developed calcinosis. They trended toward being younger at disease presentation than patients with anti-NPX2 that did not develop calcinosis (5.4/7.6 years); however, the difference was not statistically significant, but the case series is small. The mean age at disease presentation for the whole group of anti-NXP2 positive patients was 6.8 years. In a study of the UK JDM cohort in which histological heterogeneity in 101 patients with juvenile idiopathic inflammatory myopathy was studied, the mean age at disease presentation was similar to our cohort-−6.1 years. In this study, 15 patients (16.7%) were positive for anti-NXP2. On muscle biopsy, there was no specific pattern for the subgroup of patients with anti-NXP2 (12). In two other recently published studies on JDM, the mean age at presentation was higher. In the German cohort, the mean age at disease onset was 7 years, and in the Turkish study, 8.1 years (3, 4). In a German cohort, 21% of patients with anti-NXP2 were younger than 5 years of age, and it was found that patients with anti-NXP2 showed the lowest mean ever-recorded CMAS of all MSA subgroups, and almost half of them had CK levels elevated more than triple that of the normal range (4). In our cohort, 11/26 (42%) patients were younger than 5 years of age at disease onset, and in a subgroup of patients that had or developed calcinosis, 7/11 (63%) were younger than 5 years of age. So it can be assumed that the presence of anti-NXP2 in a young patient is a risk factor for worse disease course and worse outcomes. Importantly, delay in diagnosis, as an important determinant of calcinosis development, was not significantly higher in patients with calcinosis. CMAS and Kendall scores were available in only a few cases in our cohort. Many times these tests were not applicable due to the low age at disease onset. In a group with calcinosis, the Kendall test and CMAS test were available before and after treatment (8 years follow up) for only one patient who was 8 years old at disease onset (Kendall 51/71, CMAS 30/49). CK levels in our cohort of patients with calcinosis were triple that of normal range in 5/11 (45%) and 7/15 (46%) among patients without calcinosis. We did not find any significant laboratory characteristics that would discriminate among patients who had or developed calcinosis and those who did not.

The limitation of our case series study is the small number of patients and the lack of JDM patient groups without anti-NXP autoantibodies for comparison. In studies published so far, other MSA were also connected with calcinosis. In a recently published study including 58 JDM patients, 46 patients were tested for MSA; 34 (76%) had autoantibodies (3). The most commonly found autoantibodies were anti-NXP2 in 10 patients (21%), five patients (50%) developed calcinosis. MDA5 autoantibodies were found in 4 patients; 3 patients (75%) developed calcinosis. TIF1γ antibodies were found in 8 patients, four patients (50%) developed calcinosis. Among other patients with other antibodies, only 25% of patients developed calcinosis, and 25% of patients without antibodies developed calcinosis. None of the patients with Mi-2 antibodies developed calcinosis in the long-term follow-up. In a recently published German cohort, 27% of patients had calcinosis (4). Among them, 5/14 (36%) were positive for anti-NXP2, and 5/12 (42%) were positive for antiTIF1γ. So it seems that besides anti-NXP2 other autoantibodies, mainly TIF1γ, could also be connected with calcinosis.

In our cohort, patients were treated with several non-biological and biological DMARDs. We evaluated treatment by comparing the laboratory results of AST, ALT, LDH, and CK before and after treatment (mean observation time was 4.2 years). The mean values of all the parameters, as mentioned earlier, were significantly lower after treatment. However, we could not prove statistically significant differences between values before and after treatment in both subgroups of patients, with calcinosis and without calcinosis, most probably due to small numbers available for analysis. Laboratory results and muscle-function tests were not available in all patients, which is a significant limitation to our study.

A recently published study showed a significant difference in the choice of medications between pediatric rheumatologists (13). However, despite that, a high proportion of patients had the inactive disease at 2 years and had a low frequency of damage. A combination of several drugs can probably stop the progression of the disease. Although the data are very limited in our case series and the outcome of the disease was only evaluated qualitatively by the treating pediatric rheumatologist, it seemed that the most successful drug, in combination with other conventional drugs, was rituximab which was helpful in 4 difficult cases with the progression of calcinosis. Similar reports on successful treatment were also published by other authors (14–16). Recently, advances in the understanding of the immunopathology and genetics in JDM may help to specify new treatment approaches, especially when conventional therapy is not successful. An upregulated type 1 interferon signature is strongly associated with JDM, which could present the possibility for the treatment of most severe cases with JAK inhibitors (17, 18). However, the safety and efficacy of this therapy still need to be studied in clinical trials.

## Conclusion

In our case series study, 42% of JDM patients with positive anti-NXP2 presented with calcinosis or developed calcinosis during the disease course. Patients with calcinosis trended toward being younger at disease presentation and had a more severe disease course and worse outcome than patients who did not develop calcinosis despite anti-NXP. More than 60% of children who developed calcinosis in disease course were younger than 5 years of age at disease onset. There were no other significant demographical or laboratory signs that would predict the disease course and outcome. So far, there is no evidence-based treatment for calcinosis in JDM; however, the combination of several immunomodulatory and biological drugs may successfully stop the progression of calcinosis. In our study, rituximab was successfully used in stopping the progression of calcinosis in four cases.

Knowing the MSA profile in a patient with JDM is important for treatment decisions early in disease course and can contribute to more aggressive treatment in young patients with anti-NXP2.

## Data Availability Statement

The original contributions presented in the study are included in the article/supplementary material, further inquiries can be directed to the corresponding author/s.

## Ethics Statement

The studies involving human participants were reviewed and approved by Slovenian National Medical Ethics Committee. Written informed consent to participate in this study was provided by the participants' legal guardian/next of kin. Written informed consent was obtained from the minor(s)' legal guardian/next of kin for the publication of any potentially identifiable images or data included in this article.

## Author Contributions

NT and AP drafted the article. All authors contributed patients and participated in the proces of creating the final version of the article.

## Conflict of Interest

The authors declare that the research was conducted in the absence of any commercial or financial relationships that could be construed as a potential conflict of interest.

## Publisher's Note

All claims expressed in this article are solely those of the authors and do not necessarily represent those of their affiliated organizations, or those of the publisher, the editors and the reviewers. Any product that may be evaluated in this article, or claim that may be made by its manufacturer, is not guaranteed or endorsed by the publisher.
